# The Relationship Between Gut Microbiome and Bile Acids in Primates With Diverse Diets

**DOI:** 10.3389/fmicb.2022.899102

**Published:** 2022-05-11

**Authors:** Xinyue Li, Xiaochen Wang, Ziming Wang, Mingyi Zhang, Song Wang, Zuofu Xiang, Huijuan Pan, Ming Li

**Affiliations:** ^1^School of Ecology and Nature Conservation, Beijing Forestry University, Beijing, China; ^2^CAS Key Laboratory of Animal Ecology and Conservation Biology, Institute of Zoology, Beijing, China; ^3^College of Life Sciences, University of Chinese Academy of Sciences, Beijing, China; ^4^Nanning Zoo, Nanning, China; ^5^College of Life Sciences and Technology, Central South University of Forestry and Technology, Changsha, China; ^6^Center for Excellence in Animal Evolution and Genetics, Chinese Academy of Sciences, Kunming, China

**Keywords:** bile acids, diet, gut microbiome, primates, metabolomics

## Abstract

Primates have evolved a variety of feeding habits and intestinal physiological structure. Gut microbiome act as metabolic organs in many biological processes and play a vital role in adaptation to dietary niches. Gut microbiome also convert primary bile acids (BAs) to secondary. BAs profile and gut microbiome are together influenced by diets and play a significant role in nutrient absorption. The regulation between gut microbiome and BAs metabolism is bidirectional although the relationship in primates consuming diverse diets is still unclear. Here, we investigated gut microbiome structures, fecal BAs profile, and their relationship in primates preferring three distinct diets. We found that gut microbiome communities are well differentiated among dietary groups. Folivorous primates had higher Firmicutes abundance and lower *Prevotella* to *Bacaeroides* ratios, possibly related to fiber consumption. Frugivorous primates are colonized predominantly by *Prevotella* and *Bacteroides*, pointing to an increased adaptation to high-sugar and simple carbohydrate diets. Likewise, BA profiles differ according to diet in a manner predictable from the known effects of BAs on metabolism. Folivorous primates have high conjugated bile acid levels and low unconjugated to conjugated BA ratios, consistent with their fiber-rich leaf-eating diet. Much of the differentiation in secondary and unconjugated BAs is associated with microbiome composition shifts and individual bile acid concentrations are correlated with the abundance of distinct bacterial taxonomic groups. Omnivores have higher concentrations of secondary BAs, mainly lithocholic acid (LCA). These levels are significantly positively correlated with the presence of Clostrida species, showing that the digestion requirements of omnivores are different from plant-eating primates. In conclusion, gut microbiome and BAs can respond to changes in diet and are associated with nutrient component consumption in each diet primate group. Our study is the first to demonstrate BA profile differentiation among primates preferring diverse diets. BAs thus appear to work with gut microbiome to help primates adapt to their diet.

## Introduction

Over the long course of evolution primates have adapted to diverse diets and display a diverse array of anatomical specializations in their stomachs, caeca, and colons (Lambert, 1998). In addition to these structural organ changes, the set of microorganisms (commonly known as the microbiome) inhabiting gastrointestinal tracts can contribute to metabolic versatility and play an important role in host dietary adaptation, for example, digesting substrates otherwise unavailable to hosts. This can help expand host’s dietary options (Greene et al., 2020). There is mounting evidence that microbial metabolites are the major mediators of the microbiome’s influence on host physiology (Thursby and Juge, 2017; Muller et al., 2021). Bile acids (BAs), a class of metabolites produced in the liver from cholesterol and metabolized in the intestine by the gut microbiome (Wahlström et al., 2016), play an important role in lipid metabolism (De Aguiar Vallim et al., 2013). Studies have shown that the structure and function of the gut microbiome, and BAs composition all affected by diet, they play an important role in the maintenance of intestinal homeostasis and health (Clayton et al., 2018; Katsidzira et al., 2019; Trefflich et al., 2019; Ocvirk and O'Keefe, 2021). Moreover, the gut microbiome/BA profile interplay complicated by microbial biotransformation of BA types on the one hand, and the BA-mediated gut microbiome composition change on the other (Monte et al., 2009; Wahlström et al., 2016; Ocvirk and O'Keefe, 2017; Kriaa et al., 2019).

Host hepatocytes synthesize primary BAs from cholesterol (Winston and Theriot, 2020). These primary products as then conjugated with either glycine or taurine to produce conjugated BAs (Wahlström et al., 2016; Singh et al., 2019). Once host-derived primary BAs enter the gastrointestinal tract, they expedite the digestion and absorption of dietary lipids and lipophilic vitamins by forming micelles (Li and Chiang, 2014; Kiriyama and Nochi, 2019) and regulate glycolipid metabolism by activate receptors expressed in the liver and intestine, also influence immune cell function (De Aguiar Vallim et al., 2013; Fiorucci and Distrutti, 2015; Jia et al., 2019; Kiriyama and Nochi, 2019; Sato et al., 2021; Paik et al., 2022). Gut microbiome influence the composition of host BA pools by chemically modifying them into secondary BAs, mainly by deconjugation, dehydroxylation, dehydrogenation, and epimerization (Spence et al., 2006; Lucas et al., 2021). In turn, BAs also regulate the composition of gut microbiome (Winston and Theriot, 2020). The effects of BAs on gut microbiome include bacterial cell membranes damage (Begley et al., 2005; Kurdi et al., 2006), macromolecule stability disturbance (Begley et al., 2005; Merritt and Donaldson, 2009), and intracellular acidification (Kurdi et al., 2006). These important features of BAs lead to inhibition of the growth of some bacteria in the intestinal tract (Begley et al., 2005; Kurdi et al., 2006; Di Ciaula et al., 2017). Some bile acids have a variety of antibacterial activities (Begley et al., 2005; Kurdi et al., 2006). Thus, the BAs pool is a function of a collaborative metabolism of the host and the gut microbiome and the relationship between the gut microbiome and BAs is bidirectional. For example, changes in gut microbiome composition can alter BA profiles (Chen et al., 2020; Connors et al., 2020; Tanaka et al., 2020; Kamp et al., 2021). A comparative study showed that BA pools of conventional mice are more chemically diverse than gnotobiotic mouse pools (Sayin et al., 2013). BA profiles also vary according to dominant microbiome composition in the first 3 years in Japanese infants (Tanaka et al., 2020). Conversely, insufficient bile secretion, such as biliary obstruction or liver cirrhosis, leads to dramatic gut microbiome proliferation and increased bacterial translocation, highlighting bile acids’ antibacterial activity (Ding et al., 1993; Inagaki et al., 2006).

Given what we know from studies within humans, we would expect that divergence in diets among closely related species is accompanied by changes in gut microbiome and BA composition (Di Ciaula et al., 2017; Singh et al., 2019; Winston and Theriot, 2020). Primate diets include three major groups of nutrients: proteins, lipids (fats), and carbohydrates. These come from a diverse set of foods, including fruit pulp, seeds, leaves (and other structural components of plants), plant exudates, and animal matter (Lambert, 1998). These nutrition sources can be classified into three main diets: folivorous (e.g., Colobidae), omnivorous (e.g., Cercopithecinae), and frugivorous (e.g., Hylobatidae; Struhsaker, 1995). Colobines are a unique group of old world monkeys from southeast Asia and Africa who consume a folivorous diet rich in structural carbohydrates (crude fiber content up to 52%) that is difficult to digest (Lambert, 1998; Nijboer and Clauss, 2006). These primates evolved a foregut fermentation system characterized by a large, sacculated forestomach with a diverse array of microflora to digest the crude fiber (Milton, 1993; Lambert, 1998; Nijboer and Clauss, 2006), chemical secondary compounds (such as tannin, alkaloids, and terpenoids), and toxic secondary metabolites (Milton, 1993; Lambert, 1998). Sichuan snub-nosed monkeys (*Rhinopithecus roxellana*) are representative colobidae species (Jablonski and Peng, 1993). They have an unusual foregut fermentation system different from other common primates (Milton, 1993; Lambert, 1998). Macaques (*Macaca mulatta*) are omnivores and representative species of the Cercopithecinae subfamily (Fooden, 2000; Brandon-Jones et al., 2004; Sengupta and Radhakrishna, 2016). They generally feed on twigs, spears, plant seeds, herbs, fruits, leaves, and some bird eggs (Fooden, 2000). The wide food preference of macaques is one of the reasons they are widely distributed and adapted to complex habitats (Hill, 1997). Gibbons (Hylobatidae, except for *Hylobates syndactylus*), commonly known as lesser apes, are representative primate frugivores that inhabit tropical and subtropical rainforests (Chatterjee, 2009; Hon et al., 2018). Fruit is the primary component of the gibbon diet (40 ~ 78%), with high concentrations of total non-structural carbohydrates (Lambert, 1998; Kim et al., 2011; Ni et al., 2014; Dooley and Judge, 2015; Hon et al., 2018), especially fructose, as well as water and lipids (Lambert, 1998). Gibbons supplement their diets with young leaves, flowers, mature leaves, and insects (Elder, 2009). Such diversification of diet preferences by closely related species makes primates a good model for studying diet adaptation (Milton, 1993).

To elucidate the role that BAs and gut microbiome composition play in evolutionary adaptation to diverse diets, we analyzed fecal BAs and gut microbiome community profiles of representative primate species preferring one of the major diet groups. We aim to uncover correlations between BAs and the gut microbiome that accompany adaptation to specific diets.

## Materials and Methods

### Sample Collection

We collected 30 fecal samples from three primate species with different dietary preferences: the folivorous *Rhinopithecus roxellanae* (*n* = 10, Fol), the omnivorous *Macaca mulatta* (*n* = 10, Omn), and the frugivorous *Hylobates pileatus*, *Nomascus annamensis*, and *Nomascus leucogenys* (*n* = 10, Fru). Additional sample information is in the Supplementary Table S1. Fecal samples of folivorous and omnivorous primates were collected from wild groups, while frugivorous primate fecal samples were collected from captive groups because of difficulties in collecting samples in the field. All fresh fecal samples from primates were collected immediately after defecation and divided into two tubes. One contained RNA-later (Tiangen biotech, Bejing, China) for shotgun sequencing and the other was used to quantify BAs using ultra-high performance liquid chromatography coupled with tandem mass spectrometry (UHPLC–MS/MS). All samples were kept on dry ice and transported to the laboratory while frozen, where they were preserved at −80°C until processing.

### Metagenomic Community and Functional Analysis

DNA was extracted using the PowerSoil® DNA Isolation kit (MO BIO Laboratories, Carlsbad, CA, United States). DNA quality and quantity DNA were determined using a nanodrop (ND-1000) spectrophotometer (Nanodrop Technologies, Wilmington, DE, United States) and agarose gel electrophoresis. Shotgun sequencing was performed using Illumina NovaSeq 6000, with at least 10 Gb per sample (Supplementary Table S2). Adaptor and low-quality reads were trimmed using Trimmomatic (version 0.36; Bolger et al., 2014): 3′ tailing sequences were removed when the average quality over a 4-b sliding window was less than 20 and reads shorter than 70 bp were discarded. Host and human contaminations were filtered by aligning to reference genomes (*R. roxellana*: assembly ASM756505v1; *Homo sapiens*: assembly GRCh38.p13; *M. mulatta*: assembly Mmul_10; *N. leucogenys* assembly Asia_NLE_v1; and *Hylobates moloch*: assembly HMol_V2) using bowtie2 (v2.3.5; Langmead and Salzberg, 2012) with the option “–very-sensitivelocal.”

Taxonomic profiles were generated with the mOTUs profiler (v2.0.0) 52 (Milanese et al., 2019) using the following parameters: -l 75; -g 2; and -c. Single-sample metagenomic assemblies were produced with MEGAHIT (v1.2.6) (Li et al., 2015) and gene finding was performed on contigs longer than 300 bp using Prodigal. Protein sequences longer than 100 bp were functionally annotated using diamond (v0.9.24; Buchfink et al., 2015) against KEGG (v50) database (Kanehisa et al., 2016) with the parameters: -d -q -e 1e-5 -k 1.

MOTUs profiles and K-numbers were first converted to relative abundance to account for library size, then were analyzed using a nonparametric test and correlation (Supplementary Tables S4 S5). Species abundance was used to calculate alpha and beta diversities. Alpha diversity analysis was performed using observed species (Ace and Shannon index), implemented in R (vegan). Venn and principal component analyses (PCA) were performed using the OmicStudio tools.^1^ Linear discriminant analysis (LDA) effect size (LEfSe) was estimated using Galaxy.^2^

### Metabolomic Profiling

A UHPLC–MS/MS system was used to quantify BAs at Novogene Co., Ltd (Beijing, China; ExionLC™ AD UHPLC-QTRAP 6500+, AB SCIEX Corp., Boston, MA, United States). We analyzed 33 kinds of BAs along with six stable isotope-labeled internal standards obtained from ZZ Standards Co., LTD (Shanghai, China). Ammonium acetate was of analytical grade and obtained from Sigma-Aldrich (St. Louis, MO, United States). Methanol (Optima LC–MS), acetonitrile (Optima LC–MS), and formic acid (Optima LC–MS) were purchased from Thermo-Fisher Scientific (FairLawn, NJ, United States). Standard operating procedures were followed to prepare standard solutions and extract BAs from fecal samples.

Separation was performed on an Agela Venusil MP C18 column (2.1 × 100 mm, 2.5 μm) maintained at 50°C. The mobile phase, consisting of 0.1% formic acid in water (solvent A) and acetonitrile (solvent B), was delivered at a flow rate of 0.50 ml/min. The solvent gradient was set as follows: initial 20% B, 0.5 min; 20–35% B, 1 min; 35–37% B, 2.5 min; 37–38% B, 4.1 min; 38–39% B, 6 min; 39–40% B, 6.5 min; 40–44% B, 8.5 min; 44–45% B, 9 min; 45–52% B, 9.5 min; 52–55% B, 11.5 min; 55–100% B, 12.5 min; 100–20% B, 15.1 min; and 20% B, 17.1 min. The mass spectrometer was operated in negative multiple reaction mode (MRM). Parameters were as follows: IonSpray Voltage (−4,500 V), Curtain Gas (30 psi), Ion Source Temp (550°C), and Ion Source Gas of 1 and 2 (65 psi; Supplementary Table S3).

Quality control (QC) was used to evaluate the stability of methods and machines (Supplementary Table S6–1). Data files generated by HPLC-MS/MS were processed using the SCIEX OS Version 1.4 to integrate and correct peaks (Supplementary Table S6–2). Data pre-processing was done according to the “80% rule” (Bijlsma et al., 2006) and the remaining missing values were imputed using mean values (Supplementary Table S6–3). The processed data set was then entered into the SIMCA-P software package (v13.0) and was used to perform orthogonal to partial least squares-discriminate analysis (OPLS-DA).

### Statistical Analysis

Statistical analyses were performed using GraphPad Prism version 5.0 (GraphPad Software). Differences in characteristics between groups were analyzed using the Kruskal–Wallis test with Dunn *post hoc* tests (*p*_FO_, Folivorous-Omnivorous; *p*_FF_, Folivorous-Frugivorous; and *p*_OF_, Omnivorous-Frugivorous) used the adjusted *p*-value. Spearman correlations were used to explore the relationship between fecal concentrations of individual BAs metabolites and the relative abundance of bacterial taxonomic groups obtained by the LEfSe analysis, performed using OmicStudio tools. Value of *p* < 0.05 was considered statistically significant.

## Results

### Among Dietary Group Gut Microbiome Differentiation

**Figure 1:**
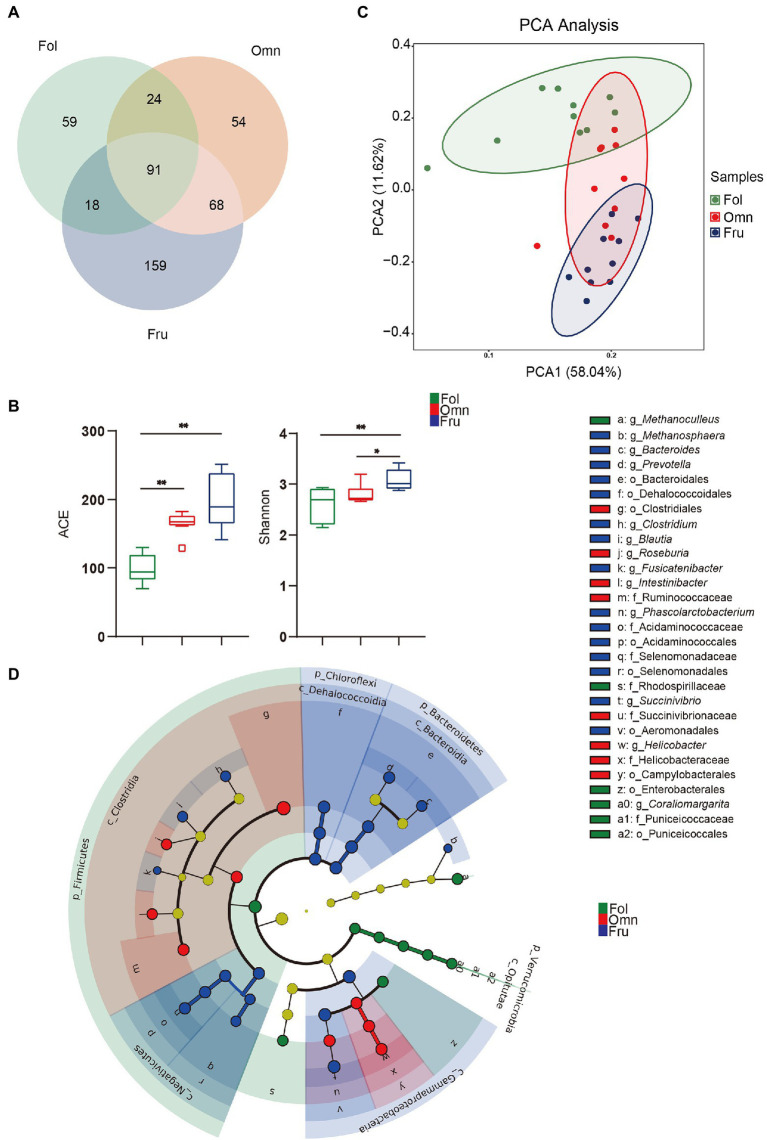
Gut microbiome structure in three primate groups. **(A)** Venn diagram at the species level. **(B)** Box-plots of alpha diversity (ACE and Shannon index) at the species level. **(C)** Microbiome beta diversity (Principal component analysis, PCA) plot among primates at the species level. **(D)** Cladogram of differential microbial prevalence identified by Linear discriminant analysis (LDA), effect size (LEfSe) analysis (LDA score > 4, one against all), from the phylum (innermost ring) to genus (outermost ring). Differentiated bacterial taxa are marked by lowercase letters. Each small circle at different taxonomic levels represents a taxon at that level, and the diameter of the circle is proportional to relative abundance. Taxa with no significant difference are yellow. Differentiated taxa are colored according to the group with the highest species abundance. Colors represent groups and nodes are colored according to the communities that play an important role in the group represented by the color. A value of *p* < 0.05 was considered statistically significant. ^*^p < 0.05; ^**^p < 0.01.

We identified 473 species from fecal samples of three dietary groups of primates. These species belong to 14 phyla, 24 classes, 38 orders, 59 families, and 110 genera (Supplementary Table S4). We found 192 species of bacteria in the folivorous (Fol) group, 237 in the omnivorous (Omn) group, and 336 in frugivorous (Fru) group (Figure 1A). Interestingly, only 91 species of bacteria are shared among all three groups, indicating appreciable species composition differentiation accompanying shifts in dietary preference (Figure 1A). Moreover, alpha diversity estimates identify significant differences among the three groups (Shannon index: *p*_FF_ = 0.0034, *p*_OF_ = 0.0412; ACE index: *p*_FF_ < 0.0001, *p*_FO_ = 0.0045; Figure 1B). Beta diversity analysis likewise shows tight, but well-separated, within-group clusters of individuals (*R* = 0.8333, *p* = 0.001; Figure 1C), reinforcing the conclusion that microbiome compositions have significantly diverged according to primate dietary preference.

We further used LEfSe to draw a cladogram that reflects differential occurrence of taxonomic terms in individual samples (Supplementary Figure S1; Figure 1D). Nine taxa are significantly more overrepresented among folivores, which belong to Firmicutes, Verrucomicrobia, Proteobacteria, and Euryarchaeota (Figure 1D; Supplementary Figure S1). Clostridia and Clostridiales are among the nine taxa preferentially found in omnivorous primates (Figure 1D; Supplementary Figure S1), while 21 taxonomic terms overabundant in frugivores belong to Bacteroidetes, Chloroflexi, Firmicutes, and Proteobacteria (Figure 1D; Supplementary Figure S1). Folivores have the highest log F/B (*p*_FF_ < 0.0001, *p*_OF_ = 0.0049) and the lowest log P/B (*p*_FO_ = 0.0008, *p*_FF_ = 0.0177; Supplementary Figures S2, S3). In contrast, omnivores and frugivores have substantially higher log P/B (*p*_FO_ = 0.0008 and *p*FF = 0.0177; Supplementary Figure S3). These results show that gut microbiome communities are influenced by host diets and may play an important role in host adaptation.

### Among Dietary Group BAs Composition Differentiation

**Figure 2:**
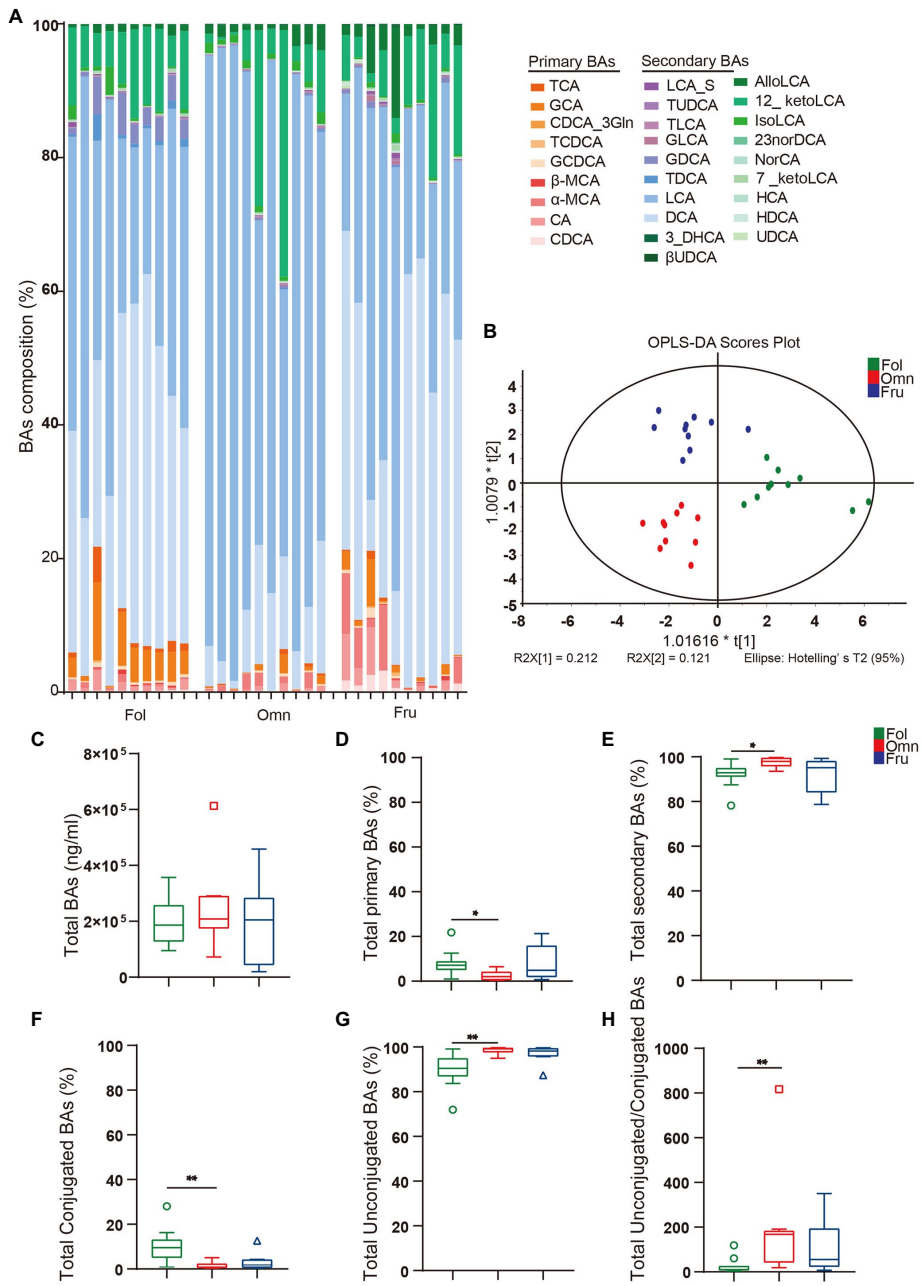
BAs metabolome. **(A)** BAs composition column. (CA, cholic acid; GCA, glycocholic acid hydrate; TCA, taurocholic acid; CDCA, chenodeoxycholic acid; GCDCA, glycochenodeoxycholic acid sodium salt; CDCA_3Gln, chenodeoxycholic acid-3-β-D-glucuronide; TCDCA, taurochenodeoxycholic acid sodium salt; DCA, deoxycholic acid; GDCA, glycodeoxycholic acid; TDCA, taurodeoxycholic acid sodium salt; UDCA, ursodeoxycholic acid; GUDCA, glycoursodeoxycholic acid; TUDCA, tauroursodeoxycholic acid dihydrate; βUDCA, 3β-ursodeoxycholic acid; LCA, lithocholic acid; TLCA, taurolithocholic acid sodium salt; GLCA, glycolithocholic acid; LCA_S, lithocholic acid 3-sulfate sodium salt; HCA, hyocholic acid; HDCA, hyodeoxycholic acid; 7_ketoLCA, 7-ketolithocholic acid; NorCA, nor cholic acid; 23norDCA, 23-nordeoxycholic acid; isoLCA, isolithocholic acid; 12_ketoLCA, 12-ketolithocholic acid; AlloLCA, allolithocholic acid; 3_DHCA, 3-dehydrocholic acid; α-MCA, alpha-muricholic acid; and β-MCA, beta-muricholic acid). **(B)** Orthogonal to partial least squares-discriminate analysis (OPLS-DA) score plots of fecal samples from the three primate dietary groups. **(C)** Total bile acids (BAs) levels. **(D)** Total primary BAs (%). **(E)** Total secondary BAs (%). **(F)** Total conjugated BAs (%). **(G)** Total unconjugated BAs (%). **(H)** The ratio of conjugated/unconjugated BAs. A value of *p* < 0.05 was considered statistically significant. ^*^p < 0.05; ^**^p < 0.01. All data are shown in box-and-whisker plots according to Tukey.

We next compared BAs profiles among groups of non-human primates consuming different diets. We observed obvious differences in BAs composition among diet groups, when viewed directly (Figure 2A) or through an OPLS-DA analysis (Figure 2B). While total BAs amounts do not appear to differ among groups (Figure 2C), we noticed differences in BAs composition (Figures 2D–H). There are more conjugated BAs (*p*_FO_ = 0.0034; Figure 2F) and a lower unconjugated to conjugated BAs ratio (*p*_FO_ = 0.0034; Figure 2H) in folivores than in other groups, while omnivores produce more total secondary BAs (*p*_FO_ = 0.0169).

**Figure 3:**
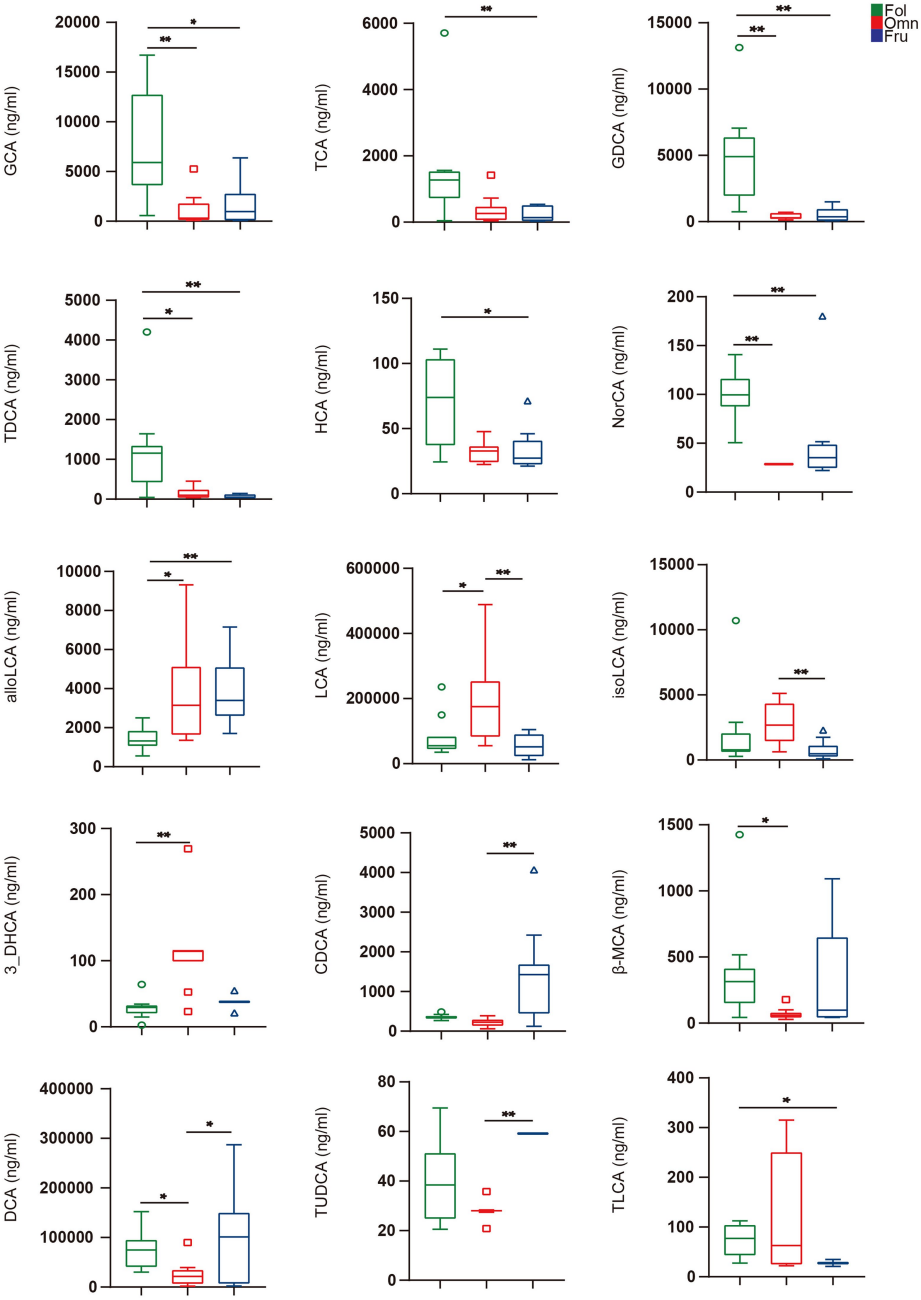
Individual BAs amounts. BAs in feces were analyzed by the Kruskal–Wallis test with Dunn *post hoc* tests (for nonparametric unpaired data) using GraphPad Prism version 5.0. A value of *p* < 0.05 was considered statistically significant. ^*^p < 0.05; ^**^p < 0.01. All data are shown in box-and-whisker plots according to Tukey.

We used the Kruskal–Wallis test with Dunn post-hoc tests to identify individual BAs whose levels differ among groups. We found that 15 such compounds (Figure 3). GCA (*p*_FO_ = 0.0026, *p*_FF_ = 0.0105), TCA (*p*_FF_ = 0.0168), GDCA (*p*_FO_ = 0.0006, *p*_FF_ = 0.0014), TDCA (*p*_FO_ = 0.0287, *p*_FF_ = 0.0010), HCA (*p*_FF_ = 0.0228), and NorCA (*p*_FO_ = 0.0003, *p*_FF_ = 0.0068) are highest in folivores, while individuals in this group are relatively deficient in alloLCA (*p*_FO_ = 0.0133, *p*_FF_ = 0.0015; Figure 3). Omnivores produce an abundance of LCA (*p*_FO_ = 0.0287, *p*_OF_ = 0.0041), isoLCA (*p*_OF_ = 0.0063), and 3_DHCA (*p*_FO_ = 0.0002), but relatively little CDCA (*p*_OF_ = 0.0002), β-MCA (*p*_FO_ = 0.0248), and DCA (*p*_FO_ = 0.0475, *p*_OF_ = 0.0412; Figure 3). Finally, frugivores produce a relatively high levels of CDCA (*p*_OF_ = 0.0002) and TUDCA (*p*_OF_ < 0.0003), but relatively little TLCA (*p*_FF_ = 0.0111; Figure 3). It thus appears that BAs composition does vary with dietary preference.

### Individual BA Concentrations Correlate With the Gut Microbiome

**Figure 4:**
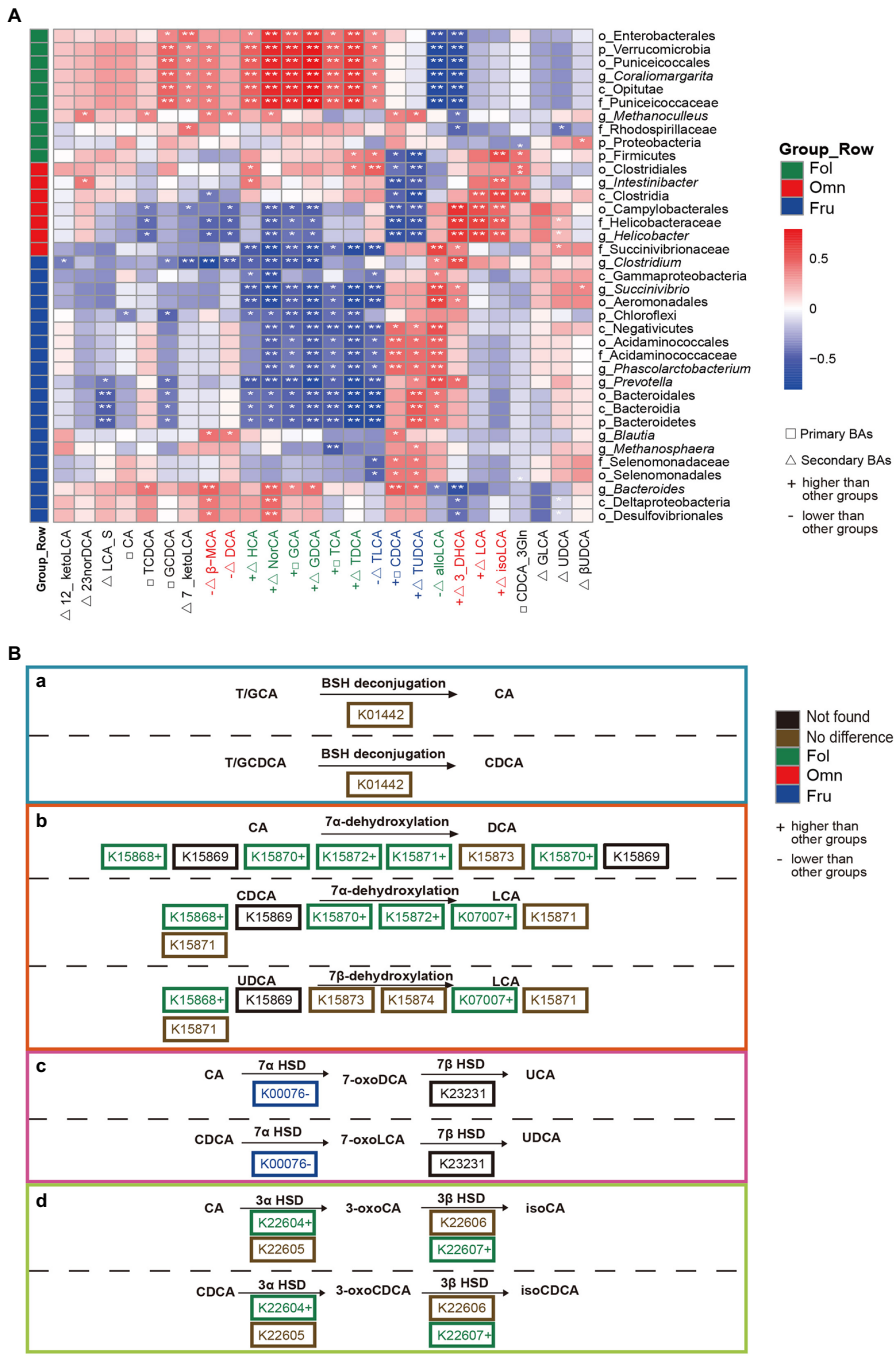
Association between BAs composition and gut microbial diversity. **(A)** Heat map summarizing the correlations of fecal bile acid concentrations and differentially abundant taxa relative abundance between groups (^*^p < 0.05; ^**^p < 0.01). **(B)** Secondary BAs metabolic pathways modified by the microbiome. (a) BSH deconjugation of T/GCA and T/GCDCA. (b) 7α/β-dehydroxylation of CA and CDCA. (c) 7α/β-dehydrogenation of CA and DCA. (d) 3α/β-dehydrogenation of CA and CDCA. The hydrolysis of taurine or glycine conjugated BAs to free BAs is performed by the bacterial bile salt hydrolase (BSH; K01442; Di Ciaula et al., 2017). In addition, following deconjugation, gut microbiota can perform 7-dehydroxylation, involving a multistep biochemical pathway found only in anaerobic gut bacteria (7-dehydroxylation: K15868, K15869, K15870, K15871, K15872, K15873, K15874, and K07007; Monte et al., 2009), bacterial dehydratases of the anaerobic flora from this region attack and remove the hydroxyl group to form 7-deoxy BAs (Monte et al., 2009), including 7α-dehydroxylation of CA and CDCA yielding DCA and LCA, respectively; BAs 7β-dehydroxylation of UDCA yielding LCA (Di Ciaula et al., 2017). Another well-recognized transformation is carried out by hydroxysteroid dehydrogenases (HSD) of intestinal bacteria (Monte et al., 2009; Ridlon et al., 2016; Lucas et al., 2021). HSDs catalyze reversible oxidation of hydroxyl groups on the C-3 (3α-HSD: K22604, K22605; 3β-HSD: K22606, K22607) and C-7 (7α-HSD: K00076; 7β-HSD: K23231) carbon positions of the bile acid steroid core (Monte et al., 2009; Ridlon et al., 2016; Lucas et al., 2021).

To investigate whether microbiome variation is associated with BAs composition, we estimated Spearman correlations within diet groups. We found 306 significant (*p* < 0.05) associations. HCA, NorCA, and most conjugated BAs (primary BAs: GCA and TCA; secondary BAs: GDCA and TDCA) are positively correlated with Enterobacterales (Proteobacteria) and Coraliomargarita (Verrucomicrobia) in folivores (Figure 4A). In contrast, alloLCA and 3_DHCA abundances are negatively correlated with the prevalence of these bacteria (Figure 4A). LCA is positively correlated with Clostridia and *Helicobacter* (Campylobacterales) in omnivores, while CDCA and TUDCA are negatively correlated with *Intestinibacter* (Clostridia) and *Helicobacter* (Campylobacterales; Figure 4A). Most conjugated BAs are negatively correlated with Chloroflexi and *Prevotella* in frugivores, whereas CDCA and TUDCA are positively correlated with *Phascolarctobacterium* abundance (Figure 4A).

To see if there is any evidence that correlations between BAs and bacterial taxon abundance may be driven by microbiome metabolism, we focused on genes in our sequence pool that may be involved in BAs metabolism. We found many genes whose products are predicted to be involved in BSH deconjugation (K01442) and 7-dehydroxylation (K15868, K15869, K15870, K15871, K15872, K15873, K15874, and K07007). We also identified genes coding for the HSD class of enzymes that catalyze the reversible oxidation of hydroxyl groups on the C-3 (3α-HSD: K22604 and K22605; 3β-HSD: K22606 and K22607) and C-7 (7α-HSD: K00076; 7β-HSD: K23231) carbon positions of the BAs steroid core. Furthermore, we found genes whose products belong to 13 KEGG ontologies involved in secondary BAs synthesis. Products of these genes cover all the major metabolic steps, except for K15869 and K23231. These were not found possibly due to detection and processing problems. Of the genes, we did identify, only three (K01442, K22605, and K07007) were detected across all samples, and one (K22607) was absent in omnivores (Supplementary Table S5). The BSH deconjugation process is both a prerequisite and a limitation for other transformation processes. There was no difference in the prevalence of the gene (K01442) that codes for BSH among dietary groups. However, we found more genes coding for 7-dehydroxylation enzymes among folivores (Figure 4B). This increase in enzyme-coding gene prevalence is broad, including K15868 (*p*_FO_ = 0.0039), K15870 (*p*_FO_ = 0.0183), K15872 (*p*_FO_ = 0.0065), and K07007 (*p*_FF_ = 0.0002, *p*_OF_ = 0.0309). K15871, K15873, and K15874 are no significantly different among dietary groups (Figure 4B). Furthermore, genes coding for 7α/β-HSD and 3α/β-HSD processing enzymes, K00076 (*p*_FF_ = 0.0021), K22604 (*p*_FO_ = 0.0436), and K22607 (*p*_FO_ = 0.0203) are also overrepresented in folivores (Figure 4B). In contrast, 7α/β-HSD coding gene K00076 (*p*_FF_ = 0.0021) is underrepresented among frugivores (Figure 4B).

## Discussion

Using microbiome shotgun sequencing and metabolite mass spectrometry, we examined gut microbiome and BAs metabolite profiles of non-human primates consuming a variety of diets. We found significant differences in both gut microbiome composition and BAs profiles among three major dietary groups (folivores, omnivores, and frugivores). Furthermore, correlations between bacterial taxon abundance and metabolite concentration vary among three groups. We further focused on the abundance of individual bacterial genes coding for BA metabolizing enzymes and found that some were associated bile acid profile differences among dietary groups. This suggests that the microbiome plays a direct role in diet-driven differentiation in BAs prevalence among primate species.

### Gut Microbiome Differentiation Among Primate Species Grouped by Dietary Preference

We found clear separation in microbial profiles among the three major dietary groups of primates, with very little overlap. This observation is consistent with previous studies that found correlations of structure and function of intestinal microbiome phylogenies and diets (Clayton et al., 2018). Firmicutes are overrepresented in folivores, along with a low *Prevotella* to *Bacaeroides* ratio. This combination is thought to promote dietary fiber digestion (Figure 1D; Supplementary Figures S1, S3; Chen et al., 2017; Nobel et al., 2018), which is important for this group since a folivorous diet includes up to 52% crude fiber (Lambert, 1998; Nijboer and Clauss, 2006). Omnivores harbor relatively more Clostrida, bacteria involved in lipid metabolism (Figure 1D; Supplementary Figure S1; Petersen et al., 2019). Both omni- and frugivores have high *Prevotella* to *Bacaeroides* ratios, suggesting a decrease in fiber digestion capacity (Supplementary Figure S3; Chen et al., 2017). High Firmicutes to Bacteroidetes ratio promote efficient energy harvesting (Supplementary Figure S2; Turnbaugh et al., 2006). It is thus unsurprising to find that omnivores and frugivores exhibit this trend. In particular, frugivores consume a diet high in non-structural carbohydrates (Lambert, 1998; Kim et al., 2011; Ni et al., 2014; Dooley and Judge, 2015; Hon et al., 2018). Consistent with this, we found that *Prevotella* (Bacteroidetes) and *Bacteroides* (Bacteroidetes) are enriched in frugivores. These bacteria help to degrade simple sugars and carbohydrates (Figure 1D; Supplementary Figure S1; Hale et al., 2019).

### Primate Diets Are Associated With Distinct Bile Microbial Transformation Profiles

We found that folivorous primates have high levels of conjugated BAs (Figure 2H). Furthermore, abundance of these metabolites (primary BAs: GCA and TCA; secondary BAs: GDCA and TDCA) were positively correlated with the prevalence of Enterobacterales (Proteobacteria) and Puniceicoccaceae (Verrucomicrobia; Figure 4A). Enterobacterales (Proteobacteria) and Coraliomargarita (Verrucomicrobia) are probably crucial intestinal flora influencing levels on the metabolic health biomarker HCA (Zheng et al., 2021). Narrow diet preference often leads to low intestinal diversity in folivorous animals. Indeed, we found that folivores in our sample exhibit lower α-diversity, possibly resulting in fewer taxa contributing *bsh* genes (Connors et al., 2020). However, we observed no difference in *bsh* gene (K01442) abundance among dietary groups (Figure 4B), showing that BAs deconjugation is not deficient in folivores despite low taxonomic diversity.

We found a positive association between LCA and Clostridia in omnivores (Figure 4A). LCA is transformed from CDCA by 7α-dehydroxylation, a multistep pathway that is highly conserved and has primarily been observed in Clostridia species (Vital et al., 2019). In addition, although increased fat intake leads to increases in LCA (Zeng et al., 2019), Clostridia can block lipid absorption and thus regulate lipid metabolism (Petersen et al., 2019). Recent studies have observed the loss of Clostridia colonization and function in individuals with metabolic syndrome and obesity. Amounts of most conjugated BAs are negatively correlated with Chloroflexi and *Prevotella* prevalence in frugivores (Figure 4A). While high-sugar diets cause metabolic disorders (Qiu et al., 2021), aggravate chronic colitis, and increase the incidence of colitis related tumors (51), *Prevotella* (Bacteroidetes) can help degrade simple sugars and carbohydrates, alleviating the adverse effects of fruit-derived sugars (Lambert, 1998; Kim et al., 2011; Ni et al., 2014; Dooley and Judge, 2015; Hon et al., 2018; Hale et al., 2019). CDCA, a metabolite associated with glucose and lipid metabolism regulation (Yu et al., 2015), is positively correlated with *Phascolarctobacterium* (Negativicutes) in frugivores. CDCA is beneficial to the host because it can prevent the growth of *Clostridioides difficile* (Nagao-Kitamoto et al., 2020), reduce inflammation, and improve gastrointestinal function (Nagao-Kitamoto et al., 2020).

Gut microbiome can carry out numerous biotransformations of BAs during their enterohepatic circulation (Ridlon et al., 2016; Singh et al., 2019). BAs metabolism by the gut microbiome mainly involve several categories of reactions, including deconjugation, dehydroxylation, dehydrogenation, epimerization, and oxidation (Monte et al., 2009; Nobel et al., 2018; Lucas et al., 2021). On the other hand, BAs have antimicrobial properties and can influence the species composition of gut microbiome (Di Ciaula et al., 2017). We found that individual bile acid and microbiome taxa are correlated, likely together playing a role in promoting nutrient absorption and metabolism, as well as maintenance of intestinal health under diverse diets.

### BA Profiles Differ According to Primate Feeding Habits

Primates have adapted to a wide variety of diets, with concomitant physiological, metabolic, and behavioral strategies to cope with difficulties presented by each feeding habit (Milton, 1993). Morphological differentiation of the digestive tract is one of the prominent adaptations driven by dietary shifts. Cercopithecine monkeys are distinguished from other primates by the presence of cheek pouches where they temporarily store and begin to digest food (Murray, 2011). Amylase found in the saliva of cercopithecine cheek pouches appears to be more active in starch digestion than the human homolog (Lambert, 1998). Colobines evolved a large sacculated forestomach with a diverse array of microbiome to digest the crude fiber in their folivorous diet (Milton, 1993; Struhsaker, 1995; Lambert, 1998). Other common primates are usually hindgut fermenters with an enlarged colon or cecum (Milton, 1993; Lambert, 1998). These two basic evolutionary strategies, morphological and behavioral, to cope with foraging difficulties are not mutually exclusive. Species vary in the extent to which they employ one or the other (Milton, 1993). For example, frugivorous and folivorous primates also supplement their diet with insects and other animal foods (Garber, 1987). This supplemental diet provides them with some of the nutrients they lack and is part of their dietary strategy (Milton, 1993).

Most previous studies have directly studied the relationship between dietary habits and gut microbiome, but rarely considered the existence of other mediating factors. We found significant differences in gut microbiome composition accompanied by shifts in BAs metabolite profiles. Folivores have more conjugated BAs (Figure 2F). These may be preferentially excreted in feces because of binding to fiber molecules before being deconjugated (Singh et al., 2019). This binding may prevent BAs re-entry into enterohepatic circulation and lead to excretion in feces (Naumann et al., 2019), thereby reducing secondary BAs production. The higher concentration of HCA in folivores indicates that these individuals are at a lower risk of metabolic problems. HCA species play critical roles in glucose homeostasis and are good predictive biomarkers of metabolic health (Zheng et al., 2021). LCA, a secondary BAs metabolite, is elevated in omnivore primates (Figure 3). LCA is one of the most hydrophobic BAs and is one of the most toxic BAs (Zheng et al., 2021). LCA accumulation is associated with increased intake of fat (Zheng et al., 2021). CDCA, a primary BAs, is prevalent in frugivorous primates (Figure 3). CDCA may be closely associated with glucose homeostasis and the metabolic status (Yu et al., 2015). Increase in CDCA levels may lead to stronger activity in regulating glucolipids and energy metabolism. Thus, CDCA elevation may be a mechanism of self-protection by frugivores in the face of a high-sugar diet (Yu et al., 2015). Gut microbiome and BAs can respond to changes in diet and are associated with fiber, lipid, sugar, and carbohydrate consumption in each diet primate group. Similar patterns have been observed in other systems. Remission induced by a therapeutic hydrolyzed protein diet is linked to improved microbiome structure in canine chronic inflammatory enteropathy, marked by decreased relative abundance of pathobionts (e.g., *Escherichia coli* and *Clostridium perfringens*), increased abundance of a secondary BAs producer (*Clostridium hiranonis*), and increased levels of secondary BAs, LCA, and DCA (Wang et al., 2019). Physiological levels of these BAs inhibit *in vitro* growth of disease-associated taxa (Wang et al., 2019). High-fat diet results in BAs profile alterations, particularly in elevated unconjugated and secondary BAs. In association with gut microbiome taxa, this elevation likely confers unfavorable impacts on colonic and host cardiometabolic health in healthy young adults (Wan et al., 2020). In addition, long-term diet influences intestinal health and correlation between BAs levels and microbiome composition may be a maintenance and regulatory mechanism of intestinal health under diverse diets (Ocvirk and O'Keefe, 2017; Oluwagbemigun et al., 2021).

The current study has several potential limitations. Stool samples were collected from different primate families, while fecal samples from frugivores were obtained from captive gibbon individuals. This forced difference in sampling may have confounding effects on our conclusions. Further experimental validation of the results in other primate populations is necessary. Our study is the first to demonstrate differences in BA profiles in primates with diverse diets. We also examine correlations between BAs and gut microbiome, highlighting a promising avenue for primate diet adaption.

## Data Availability Statement

The datasets presented in this study can be found in online repositories. The names of the repository/repositories and accession number(s) can be found at: NCBI, BioProject ID PRJNA799478 (https://www.ncbi.nlm.nih.gov/bioproject/PRJNA799478), and Supplementary Material.

## Ethics Statement

The animal study was reviewed and approved by the Committee for Animal Experiments of the Institute of Zoology (CAS).

## Author Contributions

ML and HP designed the study. XL, MZ, and SW collected samples in the field. XL and XW performed the experiments. XL, XW, and ZW analyzed the data. XL, XW, ZW, HP, and ML wrote the manuscript. ZX provided constructive comments. All authors contributed to the article and approved the submitted version.

## Funding

This study was supported by the National Natural Science Foundation of China and the Strategic Priority Research Program of Chinese Academy of Sciences (31821001, XDB31000000, and 32070404).

## Conflict of Interest

The authors declare that the research was conducted in the absence of any commercial or financial relationships that could be construed as a potential conflict of interest.

## Publisher’s Note

All claims expressed in this article are solely those of the authors and do not necessarily represent those of their affiliated organizations, or those of the publisher, the editors and the reviewers. Any product that may be evaluated in this article, or claim that may be made by its manufacturer, is not guaranteed or endorsed by the publisher.

## Footnotes

^1^ https://www.omicstudio.cn/tool

^2^ https://www.harvard.edu/

## Supplementary Material

Supplementary Table S1 Samples information.

Supplementary Table S2 Sample sequencing data evaluation.

Supplementary Table S3 Parameters of ultra-high performance liquid chromatography coupled to tandem mass spectrometry (UHPLC–MS/MS).

Supplementary Table S4 Taxonomy annotation.

Supplementary Table S5 KEGG annotation.

Supplementary Table S6 Data of ultra-high performance liquid chromatography coupled to tandem mass spectrometry (UHPLC-MS/MS). S6-1: Quality control data. S6-2: BAs quantitative results of samples. S6-3: Results of BAs data processing.

Supplementary Figure S1 Microbiome taxa with linear discriminant analysis (LDA) score greater than four of different primate groups.

Supplementary Figures S2 and S3 S2: Log Firmicutes/Bacteroidetes (Log F/B) and S3: Log *Prevotella/Bacteroides* (Log P/B) were analyzed by analyzed by the Kruskal–Wallis test with Dunn post hoc tests (for nonparametric unpaired data) using GraphPad Prism version 5.0.
