# Supplementary material for: The Relationship Between Gut Microbiome and Bile Acids in Primates With Diverse Diets
Source: Front Microbiol. 2022 May 11;13:899102. doi: 10.3389/fmicb.2022.899102 (PMC9130754; doi:10.3389/fmicb.2022.899102)
Supplement: Supplementary Figure S1 — Microbiome taxa with linear discriminant analysis (LDA) score greater than four of different primate groups. [file Data_Sheet_1.docx]

Supplementary Material

# Supplementary materials catalogue:

**Supplementary Table S1.** Samples information.

**Supplementary Table S2.** Sample sequencing data evaluation.

**Supplementary Table S3.** Parameters of ultra-high performance liquid chromatography coupled to tandem mass spectrometry (UHPLC-MS/MS).

**Supplementary Table S4.** Taxonomy annotation.

**Supplementary Table S5.** KEGG annotation.

**Supplementary Table S6.** Data of ultra-high performance liquid chromatography coupled to tandem mass spectrometry (UHPLC-MS/MS). S6-1: Quality control data. S6-2: BAs quantitative results of samples. S6-3: Results of BAs data preprocessing.

**Supplementary Figure S1.** Microbiome taxa with linear discriminant analysis (LDA) score greater than four of different primate groups.

**Supplementary Figure S2 and S3.** Log Firmicutes/Bacteroidetes (Log F/B) and S3: Log *Prevotella*/*Bacteroides* (Log P/B) were analyzed by analyzed by the Kruskal–Wallis test with Dunn post hoc tests (for nonparametric unpaired data) using GraphPad Prism version 5.0.

| **Group** | **Species** | **Sampling location** | **Type** | **Sample size** | **Sample ID** |
| --- | --- | --- | --- | --- | --- |
| Folivorous (Fol) | *Rhinopithecus roxellanae* | Shennongjia National Park, HuBei, China | Wild | 10 | Fol1  Fol2  Fol3  Fol4  Fol5  Fol6  Fol7  Fol8  Fol9  Fol10 |
| Omnivorous (Omn) | *Macaca mulatta* | Shennongjia National Park, HuBei, China | Wild | 10 | Omn1  Omn2  Omn3  Omn4  Omn5  Omn6  Omn7  Omn8  Omn9  Omn10 |
| Frugivorous (Fru) | *Hylobates pileatus* | Nanning zoo, guangxi China | Captive | 10 | Fru1  Fru8  Fru3  Fru4  Fru6  Fru2  Fru5  Fru7  Fru9  Fru10 |
|  | *Nomascus leucogenys* |  |  |  |  |
|  | *Nomascus annamensis* |  |  |  |  |

**Supplementary Table S1 Samples information**

**Supplementary Table S2. Sample sequencing data evaluation**

| **Samples** | **Reads** | **Data(G)** | **Q20(%)** | **Q30(%)** |
| --- | --- | --- | --- | --- |
| **Fol1** | 34519662 | 10.3 | 97.41 | 92.92 |
| **Fol2** | 33629956 | 9.98 | 97.51 | 93.16 |
| **Fol3** | 36496550 | 10.9 | 97.45 | 93.01 |
| **Fol4** | 40433713 | 12.09 | 96.96 | 91.95 |
| **Fol5** | 36277666 | 10.84 | 97.52 | 93.19 |
| **Fol6** | 33359904 | 9.95 | 97.52 | 93.18 |
| **Fol7** | 34054136 | 10.09 | 97.44 | 93.05 |
| **Fol8** | 33658155 | 10.04 | 97.41 | 92.95 |
| **Fol9** | 42143028 | 12.57 | 97.61 | 93.32 |
| **Fol10** | 36945976 | 11.03 | 97.62 | 93.34 |
| **Omn1** | 33619794 | 10.06 | 97.74 | 93.46 |
| **Omn2** | 35787186 | 10.69 | 97.5 | 92.88 |
| **Omn3** | 34791271 | 10.38 | 97.66 | 93.37 |
| **Omn4** | 37001442 | 11.01 | 97.6 | 93.27 |
| **Omn5** | 42,108,253 | 12.59 | 97.48 | 93.23 |
| **Omn6** | 35,888,896 | 10.74 | 97.45 | 93.2 |
| **Omn7** | 33,474,325 | 10.02 | 97.37 | 93.09 |
| **Omn8** | 33,732,323 | 10.06 | 97.68 | 93.57 |
| **Omn9** | 35,394,909 | 10.58 | 97.5 | 93.28 |
| **Omn10** | 34,034,369 | 10.17 | 97.5 | 93.27 |
| **Fru1** | 33,577,496 | 10.04 | 97.83 | 94.01 |
| **Fru2** | 37,041,006 | 11.06 | 98.08 | 94.46 |
| **Fru3** | 35,243,892 | 10.54 | 97.83 | 93.97 |
| **Fru4** | 34,013,205 | 10.16 | 97.75 | 93.87 |
| **Fru5** | 33,707,492 | 10.08 | 97.75 | 93.72 |
| **Fru6** | 34,079,821 | 10.17 | 97.91 | 94.18 |
| **Fru7** | 33,688,243 | 10.08 | 97.55 | 93.37 |
| **Fru8** | 37,965,144 | 11.34 | 97.88 | 94.16 |
| **Fru9** | 34,422,488 | 10.28 | 97.9 | 94.11 |
| **Fru10** | 34,226,156 | 10.22 | 97.97 | 94.33 |

Notes：Samples: sample number; Reads: reads number; Data: sequencing data volume; Q20(%): the percentage of bases with Quality Score greater than or equal to 20 in the total number of bases; Q30(%): the percentage of bases with Quality Score greater than or equal to 30 in the total number of bases.

**Supplementary Table S3.** **Parameters of UHPLC-MS/MS**

| **NO.** | **Component Name** | **QI** | **Q3** | **RT (min)** | **DP** | **CE** |
| --- | --- | --- | --- | --- | --- | --- |
| **1** | THDCA (Taurohyodeoxycholic acid sodium salt) | 498.3 | 498.3 | 3.5 | -35 | -20 |
| **2** | THCA (Taurohyocholicacidsodium salt) | 514.3 | 514.3 | 2.94 | -90 | -15 |
| **3** | 7 _ketoLCA (7-Ketolithocholic acid) | 389.3 | 389.3 | 9.08 | -125 | -15 |
| **4** | NorCA (Nor Cholic acid) | 393.2 | 393.2 | 4.39 | -90 | -45 |
| **5** | TDCA (Taurodeoxycholic acid sodium salt) | 498.2 | 498.2 | 7.09 | -47 | -20 |
| **6** | HDCA (Hyodeoxycholic acid) | 391.3 | 391.3 | 7.67 | -90 | -23 |
| **7** | GHCA (Glycohyocholic acid sodium salt) | 464.2 | 464.2 | 3.32 | -20 | -15 |
| **8** | 23norDCA (23-Nordeoxycholic acid) | 377.2 | 377.2 | 9.42 | -90 | -15 |
| **9** | IsoLCA (Isolithocholic acid) | 375.3 | 375.3 | 12.94 | -85 | -25 |
| **10** | 12_ ketoLCA (12-ketolithocholic acid) | 389.3 | 389.3 | 9.56 | -80 | -22 |
| **11** | AlloLCA (Allolithocholic acid) | 375.3 | 375.3 | 12.8 | -65 | -20 |
| **12** | LCA_S (Lithocholic acid 3-sulfate sodium salt) | 455.3 | 455.3 | 12.3 | -50 | -20 |
| **13** | DCA (Deoxycholic acid) | 391.3 | 391.3 | 11.12 | -65 | -26 |
| **14** | GLCA (Glycolithocholic acid) | 432.3 | 432.3 | 11.54 | -53 | -20 |
| **15** | TCDCA (Taurochenodeoxycholic acid sodium salt) | 498.2 | 498.2 | 6.17 | -20 | -45 |
| **16** | GUDCA (Glycoursodeoxycholic acid) | 448.2 | 448.2 | 4.1 | -43 | -20 |
| **17** | CDCA (Chenodeoxycholic acid) | 391.2 | 391.2 | 10.76 | -42 | -20 |
| **18** | CA (Cholic acid) | 407.3 | 407.3 | 6.75 | -87 | -25 |
| **19** | TUDCA (Tauroursodeoxycholic acid Dihydrate) | 498.2 | 498.2 | 3.56 | -80 | -25 |
| **20** | CDCA_3Gln (Chenodeoxycholic acid-3-β-D-glucuronide) | 567.2 | 567.2 | 6.81 | -65 | -20 |
| **21** | LCA (Lithocholic acid) | 375.3 | 375.3 | 13.06 | -86 | -16 |
| **22** | Βudca (3β-Ursodeoxycholic acid) | 391.3 | 391.3 | 6.81 | -95 | -15 |
| **23** | GCDCA (Glycochenodeoxycholic acid sodium salt) | 448.3 | 448.3 | 7.39 | -40 | -20 |
| **24** | GCA (Glycocholic acid hydrate) | 464.3 | 464.3 | 4.02 | -90 | -22 |
| **25** | TLCA (Taurolithocholic acid sodium salt) | 482.2 | 482.2 | 11.14 | -25 | -20 |
| **26** | UDCA (Ursodeoxycholic acid) | 391.3 | 391.3 | 7.46 | -85 | -25 |
| **27** | 3_DHCA (3-Dehydrocholic acid) | 405.2 | 405.2 | 6.33 | -70 | -25 |
| **28** | HCA (Hyocholic acid) | 407.3 | 407.3 | 5.66 | -45 | -20 |
| **29** | α-MCA (Alpha-Muricholic acid) | 407.3 | 407.2 | 4.32 | -90 | -20 |
| **30** | β-MCA (Beta-Muricholic acid) | 407.3 | 407.1 | 4.77 | -135 | -20 |
| **31** | TCA (Taurocholic acid) | 514.2 | 514.2 | 3.64 | -75 | -20 |
| **32** | T-α-MCA (Tauro-alpha-Muricholic acid sodium salt) | 514.2 | 514.1 | 2.44 | -50 | -20 |
| **33** | GDCA (Glycodeoxycholic acid) | 448.3 | 448.3 | 8.11 | -60 | -20 |
| **34** | GCA-d4 | 468.3 | 468.3 | 3.99 | -80 | -20 |
| **35** | CA-d4 | 411.3 | 411.3 | 6.68 | -80 | -15 |
| **36** | UDCA-d4 | 395.3 | 395.3 | 7.42 | -90 | -15 |
| **37** | GCDCA-d4 | 452.2 | 452.2 | 7.34 | -85 | -20 |
| **38** | CDCA-d4 | 395.3 | 395.2 | 10.74 | -140 | -15 |
| **39** | LCA-d4 | 379.3 | 379.3 | 13.03 | -95 | -15 |

Notes: Q1: precursor ion; Q3: daughter ion; RT: retention time; DP: declustering potential; CE: collision energy


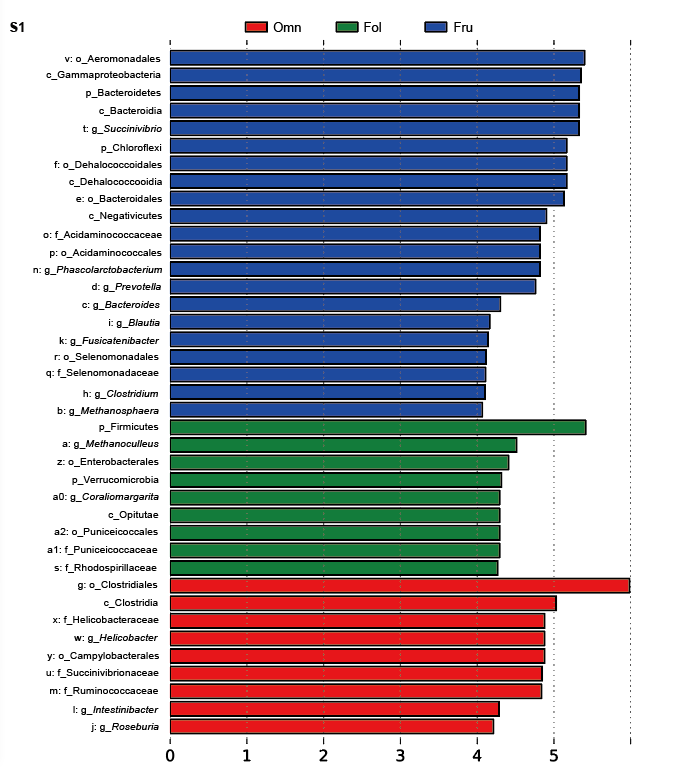


Supplementary Figure S1: Microbiome taxa with linear discriminant analysis (LDA) score greater than four of different primate groups.


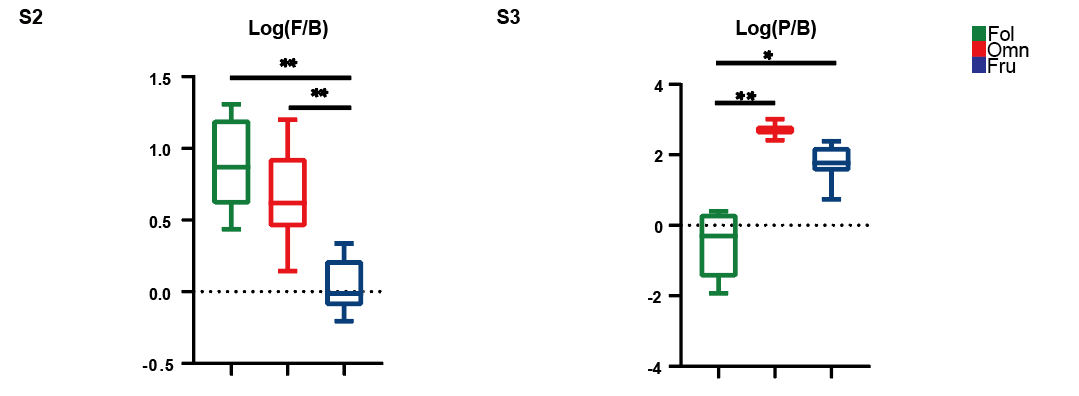


Supplementary Figure S2: Log Firmicutes/Bacteroidetes (Log F/B) and S3: Log *Prevotella*/*Bacteroides* (Log P/B) were analyzed by the Kruskal–Wallis test with Dunn post hoc tests (for nonparametric unpaired data) using GraphPad Prism version 5.0. A *p*-value < 0.05 was considered statistically significant. * p < 0.05, ** p < 0.01. Data are shown in box-and-whisker plots according to Tukey.
